# Priming from within: TLR2 dependent but receptor independent activation of the mammary macrophage inflammasome by Streptococcus uberis

**DOI:** 10.3389/fcimb.2024.1444178

**Published:** 2024-10-11

**Authors:** Abbie Hinds, Philip Ward, Nathan Archer, James Leigh

**Affiliations:** ^1^ School of Veterinary Medicine and Science, University of Nottingham, Nottingham, United Kingdom; ^2^ Department of Infection Biology and Microbiomes, University of Liverpool, Cheshire, United Kingdom; ^3^ The Division of Structural Biology (STRUBI) for Genomic Medicine, Oxford, United Kingdom

**Keywords:** *Streptococcus*, mastitis, inflammasome, TLR2, NLRP3

## Abstract

**Introduction:**

*Streptococcus uberis* is a member of the pyogenic cluster of *Streptococcus* commonly associated with intramammary infection and mastitis in dairy cattle. It is a poorly controlled globally endemic pathogen responsible for a significant cause of the disease worldwide. The ruminant mammary gland provides an atypical body niche in which immune cell surveillance occurs on both sides of the epithelial tissue. *S. uberis* does not cause disease in non-ruminant species and is an asymptomatic commensal in other body niches. *S. uberis* exploits the unusual niche of the mammary gland to initiate an innate response from bovine mammary macrophage (BMMO) present in the secretion (milk) in which it can resist the host immune responses. As a result – and unexpectedly - the host inflammatory response is a key step in the pathogenesis of *S.uberis*, without which colonisation is impaired. In contrast to other bacteria pathogenic to the bovine mammary gland, *S. uberis* does not elicit innate responses from epithelial tissues; initial recognition of infection is via macrophages within milk.

**Methods:**

We dissected the role of the bacterial protein SUB1154 in the inflammasome pathway using *ex vivo* bovine mammary macrophages isolated from milk, recombinant protein expression, and a panel of inhibitors, agonists, and antagonists. We combine this with reverse-transcription quantitative real-time PCR to investigate the mechanisms underlying SUB1154-mediated priming of the immune response.

**Results:**

Here, we show that SUB1154 is responsible for priming the NLRP3 inflammasome in macrophages found in the mammary gland. Without SUB1154, IL-1β is not produced, and we were able to restore IL-1β responses to a sub1154 deletion *S. uberis* mutant using recombinant SUB1154. Surprisingly, only by blocking internalisation, or the cytoplasmic TIR domain of TLR2 were we able to block SUB1154-mediated priming.

**Discussion:**

Together, our data unifies several contrasting past studies and provides new mechanistic understanding of potential early interactions between pyogenic streptococci and the host.

## 1 Introduction

*Streptococcus uberis* is a poorly controlled cause of intramammary infection in dairy cattle, responsible for a high proportion of bovine mastitis worldwide. It is the leading cause of the disease in the UK (Bradley et al., 2007) and is a predominant cause in other developed dairy industries. Poor control of intramammary infection with this organism stems from its route of transmission from the environment seeded by asymptomatic carriage within the gut (Davies et al., 2016). Consequently, infection can be considered opportunistic and correspondingly the population of *S. uberis* isolated from the disease is diverse (Davies et al., 2016; Whiley et al., 2024).

Following entry of *S. uberis* into the lactating bovine mammary gland, colonisation is dependent on replication in the extracellular environment (Smith et al., 2003). Virulent strains *in vivo* can reach in excess of 10^6^-10^7^ cfu/ml in milk (Leigh et al., 2010; Egan et al., 2012; Tassi et al., 2013; Hossain et al., 2015). Less virulent, or attenuated strains, colonise less well (~10^3^-10^4^ fold). Recent studies have shown that colonisation and virulence are substantially reduced by deletion of *sub1154;* encoding a cell envelope (sortase anchored) serine protease (Archer et al., 2020).

*S. uberis* fails to induce innate responses directly from mammary epithelial cells (MEC). Moyes et al., 2010, showed the responses from MEC obtained during intramammary infection of cattle were consistent with stimulation by activated macrophages rather than direct stimulation by bacteria. Consistent with this observation, Günther et al., 2016a, subsequently demonstrated *in vitro* that *S. uberis* failed to induce innate responses in primary MEC, but did induce responses from macrophages derived from blood, an observation subsequently reproduced in macrophages obtained from bovine milk (Archer et al., 2020). The use of primary *ex vivo* macrophages from the bovine mammary gland has been difficult, hence the use of cells lines and blood derived and differentiated macrophages previously. However, we recently developed and tested a method which reproducibly obtains reliable macrophage preparations from bovine milk. Through standardisation of the macrophage response to a known stimulant, analysis of host-pathogen interactions across multiple experiments was possible (Tomes et al., 2024). This has enabled the use of macrophages obtained directly from the mammary gland for such investigations (Tomes et al., 2024) and is deployed in the present work to describe the unusual aetiology of *S. uberis* pathogenesis.

Our earlier work found that srtA mutated *S. uberis* could only transiently infect the mammary gland (Leigh et al., 2010). In the same work, deletion of several sortase-anchored proteins, such as SUB1154 also severely restricted its ability to colonise and cause disease (Leigh et al., 2010). Our subsequent work demonstrated that while *in vivo* mammary gland challenge with wildtype *S. uberis* induced the production of CXCL8, IL-6, and IL-1β and clinical disease, deletion of SUB1154 ablated these host responses. Truncating the protein to prevent anchoring to the bacterial cell wall attenuated, but did not eliminate the inflammasome response. Paradoxically, we found that only when *S. uberis* could induce the host immune response could it colonise the mammary gland. Instead, colonisation correlated well with the increasing BSA concentrations found in the inflamed mammary gland. We demonstrated *ex-vivo* that the host IL-1β release from BMMOs depends on SUB1154 and involves Caspase 1 and NLRP3 oligomerisation. Overall, we described a model in which SUB1154 enables *S. uberis* to elicit the inflammasome response and exploit the local, host damage caused by these inflammatory response [to which *S. uberis* is resistant in the presence of milk (Leigh et al., 1990; Hill et al., 1994)].

Activation of the inflammasome in bovine mammary macrophages (BMMOs) and the production of the pro-inflammatory cytokine interleukin-1β (IL-1β) is dependent on the SUB1154 protein (Archer et al., 2020). Unexpectedly, this host response coincides with sustained, high-level colonisation. This paradox was reconciled through a model in which the pathogen resists the induced host defences. These responses damage host tissue, increasing nutrient availability, which enhances bacterial growth (Archer et al., 2020). This pathogenesis, in which bacterial virulence is enhanced by the local innate host response, although unusual, is not unique; inflammasome driven host responses have been associated with enhancing colonisation during other bacterial (streptococcal) infections (Günther et al., 2016a; LaRock et al., 2020). Together, this suggests that the induction of the inflammasome is a key aspect of the early pathogenesis of bovine mastitis resulting from intramammary infection by *S. uberis*.

Pathogens are typically detected by the host via pattern recognition receptors (PRRs). These recognise pathogen-associated molecular patterns (PAMPs) (von Moltke et al., 2013; Wang et al., 2014; Chen et al., 2017). This initiates a signalling cascade, leading to the formation of the inflammasome and expression of inactive pro-inflammatory cytokines such as pro-IL-1β and pro-IL-18 (Sharma and Kanneganti, 2016). Following this initial priming event, the host inflammatory responses are activated through the recruitment of caspase to the inflammasome. This two-step model has been described widely (Sharma and Kanneganti, 2016). Several inflammasome complexes have now been characterised, including NLRP1, NLRP3, NLRC4, pyrin, and AIM2, with evidence emerging of further complexes such as NLRP6 and IFI16 (Guo et al., 2015). Recruitment of caspases to these complexes to cause or effect cleavage of pro-IL-1β and pro-IL-18 has typically been linked to the presence of specific pathogen or damage associated molecular patterns (PAMPs or DAMPs) such as extracellular ATP, particulate crystals (Bauernfeind et al., 2009) or the M protein of *S. pyogenes* (Rathinam and Fitzgerald, 2016; Valderrama et al., 2017). Release of pro-inflammatory cytokines can result in maturation of inflammasomes in nearby cells, thereby amplifying the local inflammatory response.

Recently, we developed an improved model for isolation and interrogation of ex-vivo BMMOs (Tomes et al., 2024). In the present work, we exploit this to further elucidate the mechanism by which *S. uberis* and the cell envelope serine protease, SUB1154, mediates host production of inflammatory cytokines in bovine mammary macrophages in the earliest stages of infection.

## 2 Materials and methods

### 2.1 Isolation of BMMOs from milk

Raw milk was collected from bulk tank at the University of Nottingham, Sutton Bonington campus dairy centre. The somatic cell count (SCC) was determined using a DeLaval Cell Counter (DCC). Clinical infection within the herd was defined as >200 cells/µL; milk would be discarded as macrophages may have already been primed and activated which would directly affect subsequent results. A SCC of <200 cells/µL was therefore the optimal reading for experimental progression. BMMOs were isolated from milk following the protocol described in Tomes et al., 2024.

### 2.2 Bacterial strains and culturing conditions

*S. uberis* strains were cultured in Brain Heart Infusion (BHI) media (Oxoid, CM1135) at 37°C overnight. Bacterial cultures were washed 3 times in PBS at 5000xg for 3 min and then heat-killed at 63°C for 30 min. Cultures were resuspended in IMDM at an optical density of 1 (2x10^7^/mL) at 600 nm wavelength. *S. uberis* strain 0140J (strain ATCC BAA-854/0140J), originally isolated from a clinical case of bovine mastitis in the UK, was used throughout this study as a reference strain. The SUB1154 deletion mutant (0140JΔ*sub1154*) was generated as previously described by allelic exchange mutagenesis (Leigh et al., 2010).

### 2.3 Recombinant SUB1154 protein purification

Recombinant SUB1154 protein (rSUB1154; Glu61-Thr1113-Arg-Ser 6[His]-TAA) was constructed to accommodate predicted propeptide processing/autocatalytic cleavage at the N-terminus as described for C5a peptidase from *Streptococcus pyogenes* (Anderson et al., 2002). The C-terminus of the recombinant matched the predicted sortase-cleaved product leading into a 6[His] affinity tag. The proteolytically compromised (rSUB1154NP) construct with mutation Ser496Ala was engineered from rSUB1154 by reverse PCR with the aim of disrupting the Ser, His, Asp catalytic triad common to most serine proteases. The sense and antisense Ser mutation oligonucleotide primers used were 5’-CAAAGATGTCAGGAACTGCTGCTGCAAGTCC and 5’-GCATGTGGACTTGCAGCAGCAGTTCCTGACATC respectively.

The rSUB1154 and rSUB1154NP proteins were purified from *E. coli* hosts using the pQE-1 expression plasmid containing an ampicillin resistance marker gene, a T5 promoter and a LacO operon. *E. coli* host strains were inoculated in Luria-Bertani (LB) broth (Sigma, 51208) with 50 µg/mL of ampicillin (Sigma, A9518) and incubated overnight at 37°C. The inoculate was then diluted 1:50 with 100 mL prewarmed LB broth plus ampicillin in a 500 mL conical flask and grown in a shaking incubator (GFL Incshaker, T3031) for 3h at 160 RPM and 37°C. 1 mM of isopropyl β-D-1-thiogalactopyranoside (IPTG) (Sigma, I6758) was added and incubation was continued for a further 2h. Cells were then harvested by centrifugation at 4500xg for 15 min at 4°C. The cells were washed in sterile PBS and the pellets were frozen at -20°C overnight.

The harvested bacterial cells were thawed on ice and resuspended in 20 mL of CelLytic™ B Cell Reagent (Sigma, B7435) per gram of cell pellet. Proteolytic degradation was prevented by the addition of 1 mM cOmplete™ protease inhibitors (Roche, 11697498001). To completely lyse the bacterial cells the suspension was incubated shaking at 160 RPM at room temperature for 15 min and any debris was removed by centrifugation at 16,000xg for 10 min. The supernatants were collected and centrifuged at 16,000xg for 10 min. The resulting supernatants were filter sterilised using a 0.45 µM filter (Millex^®^HA, SLHAM33SS) and purification of the recombinant proteins achieved using HisPur™ Ni-NTA Chromatography protein purification cartridges (Thermo Scientific, 90098) following the manufacturer’s instructions. Briefly, at a flow rate of 1 mL/min, the columns were flushed with 10 mL of deionised water to remove the storage solution and equilibrated with 10 mL of wash buffer (10 mM imidazole (Sigma, I5513-5G) in PBS). After loading the filtered supernatants, unbound proteins were removed using 10 mL of wash buffer and the target proteins eluted using elution buffer (250 mM imidazole in PBS). The eluants were collected in 1 mL fractions and those containing the SUB1154 proteins were determined by SDS-PAGE.

Removal of imidazole was achieved by dialysis using Slide-A-Lyzer dialysis cassettes (Thermo Scientific, 2160728) following the manufacturer’s instructions. Cassette membranes were hydrated in 1L of ultrapure water for 2 min. Fractions containing the soluble rSUB1154 proteins were added to the cassettes and suspended, gently stirring, in the water for ~20h at 4°C with two changes of water at 2h and 4h. Purified rSUB1154 proteins were extracted from the dialysis cassette and any endotoxin was removed using Pierce^®^ High-Capacity Endotoxin Removal Spin Columns (0.50 mL Thermo Scientific, 88274). Columns were washed with ultrapure water and then equilibrated with endotoxin free PBS. Purified rSUB1154 proteins were added to the columns at a flow rate of 10-15 mL/h. An additional 1 mL of PBS was put through the pump and 1 mL directly to the resin to ensure elution of proteins. Concentration of the rSUB1154 proteins were determined using a spectrophotometer/fluorometer (Denovix, DS-11 FX+) and stored with 10% glycerol (Fisher Chemical, G/0650/08) at -80°C.

### 2.4 SDS-PAGE

Samples were mixed 1:1 with (2X) SDS sample buffer (Novex, 2201443) and heated to 95°C for 5 min. Samples and blue prestained protein standard, broad range ladder (11-250 kDa; New England Biolabs, P7718S) were loaded onto 10% Mini-PROTEAN TGX stain-free gels (Biorad, 4568036) with running buffer containing 25 mM Tris, 200 mM glycine (Thermo Fischer, 28363) and 0.1% SDS and electrophoresis conducted at 120 V. Gels were exposed to UV for 5 min and image captured using a Biorad ChemiDoc™ Imaging System.

### 2.5 BMMO challenge

Following isolation, BMMOs were challenged with various stimuli for 20h. We previously found (Tomes et al., 2024) that heat-killing *S. uberis* did not significantly perturb the macrophage response, but enabled us to have fine control over the multiplicity of infection in our model. Heat-killed *S. uberis* strains at a multiplicity of infection (MOI) of 50:1 bacterium:BMMO; 10 ng/mL LPS (isolated from *E. coli* 0111:B4, Millipore, LPS25); 2 nM rSUB1154/NP; inflammasome primer 1.0 µg/mL Pam3CSK4 (Tocris, 4633); inflammasome activator 500 µg/mL silica (Sigma, 421553); cell entry inhibitor 10 µM Cytochalasin D (CyD) (Tocris, 1233) for 2h prior to additional stimuli for 20h; TLR2 inhibitor 100 µM C29 (Adooq Bioscience, A17160) for 1h prior to additional stimuli for 20h.

### 2.6 ELISA

Bovine IL-1β was detected by ELISA using the Invitrogen Reagent Kit (ESS0027) following manufacturer’s instructions. Coating antibody was diluted in BupH Carbonate/Bicarbonate Buffer (0.2 M, Invitrogen, 28382) and incubated overnight at room temperature in 96-well plates (Thermo Scientific, clear flat-bottom immune nonsterile, 3355). Plates were aspirated and incubated for 1h at room temperature in blocking buffer (4% BSA and 5% sucrose (Sigma, S0389) in PBS). Wells were washed with PBS + 0.05% Tween-20 (Sigma, P1379) and the detection antibody and streptavidin-HRP (horseradish peroxidase) were diluted in reagent diluent (4% BSA in PBS).

Absorbance was measured at wavelengths of 450nm and 550nm using a Varioskan^®^ Flash multimode plate reader (Thermo Scientific). Optical imperfections were corrected for by subtracting the 550nm reading from the 450nm. A standard curve, using either second order polynomial (quadratic) or third order polynomial (cubic), was generated and sample IL-1β concentrations were interpolated. Anything returning below 1 (i.e. negative due to assay sensitivity) was considered 0.

### 2.7 RNA extraction and real-time quantitative reverse transcription polymerase chain reaction

RNA was extracted from BMMOs using the Qiagen RNeasy Mini Kit (74104). RNeasy lysis buffer and 70% ethanol in equal volumes were added to the BMMOs and centrifuged in spin columns at 8,000xg for 15 sec. The membrane-bound RNA was washed in RW1 buffer, and the washing buffer removed by centrifugation (8,000xg for 15 sec). RNA was then washed with RPE buffer twice and the washing buffer removed by centrifugation (8,000xg for 15 sec and subsequently 2 min) prior to elution of the RNA in RNase free water. RNA was precipitated by the addition of 0.1 volume of sodium acetate (Sigma, W302406), 3 volumes of ethanol and 2 µL of glycoblue coprecipitate (Invitrogen, AM9515) and incubation overnight at -20°C. Precipitated RNA was collected by centrifugation (15,000xg; 15 min), washed in 70% ethanol and pelleted by centrifugation at 15,000xg for 3 min. The RNA pellets were subsequently dried, resuspended in RNase free water and the concentration determined and adjusted to approximately 15 ng/µL using a spectrophotometer/fluorometer (Denovix, DS-11 FX+).

RT qRT-PCR of the RNA was completed using Luna^®^ Universal One-Step RT-qPCR Kit with the primers (Sigma) listed in **Table 1** (10 µM in DNase free water). 20 µL reactions were performed in microamp optical 96-well reaction plates (Applied Biosystems, N8010560) on a Biorad CFX connect real-time system instrument (788BR7113). Biorad CFX maestro software was used to complete the SYBR^®^ scan mode protocol using the thermocycling protocol below (**Table 2**).

**Table 1:** RT qRT-PCR RNA primers.

Gene	Sequence	Function	Forward Primer (5’-3’)	Reverse Primer (5’-3’)	Tm (°C)	Product Size (bp)	Gene size (bp)
*Actin Beta (* ***ACTB*** *)*	*Bos taurus* NM_173979.3	Part of the actin cytoskeleton; involved in cell shape and movement.	GCAGGAGTACGATGAGTCCG	TGTCACCTTCACCGTTCCAG	64.8 66.3	220	1948
*Glyceraldehyde 3-phosphate dehydrognase (* ***GAPDH*** *)*	*Bos taurus* NM_001034034.2	Enzyme involved in the breakdown of glucose.	GCCCTCTCAAGGGCATTCTA	ATTCTCAGTGTGGCGGAGAT	65.4 63.7	297	1279
*Ribosomal Protein 13 (* ***RPL13a*** *)*	*Bos taurus* NM_001015543.2	Component of the 60S ribosomal subunit.	GGCTCGCAAGATCCGTAGAC	ACAGGATAAGCTTGGAGCGG	66.0 65.4	293	926
*Toll-like Receptor 2 (* ***TLR2*** *)*	*Bos taurus* NM_174197.2	Pathogen recognition and activation of innate immunity.	TGTAAACTTGAGAGTGGAGGTCA	TTTCACACCTGCCGTGAGAC	62.6 66.4	200	3513
*Nuclear Factor Kappa B (* ***NF-κB*** *)*	*Bos taurus* NM_001192970.1 REL proto-oncogene, NF-κB subunit	Transcriptional regulator of the immune response.	ATACCTGCCAGATGAAAAGGACAC	TCGGTAGCATGGCTGAAGCAG	66.1 69.6	296	1830
*Pro-Caspase-1 (* ***CASP1*** *)*	*Bos taurus* XM_002692921.5 Predicted transcript variant X2	Proteolytically cleaves precursor forms of IL-1β and IL-18.	GGAAGCCATGGCCGACAA	GCCAGGTGGGAGTCTTCTTC	69.6 65.1	258	2002
*Interleukin-1 beta Precursor (* ***Pro-IL-1β*** *)*	*Bos taurus* NM_174093.1	Cytokine that mainly mediates the inflammatory response but is also involved in cell proliferation, differentiation, and apoptosis. Cleaved to active form by CASP1.	CGACTGCCTTCCCTGCATTA	TTTTTCACAGATGCGCCTGC	67.3 68.4	294	1736

All primers were supplied by Sigma-Aldrich and were designed using the mRNA sequence on the NCBI database. The first three rows are the reference genes, and the following four rows are the target genes. The melting temperature (Tm) for the forward primer is the first value and the reverse primer is the second value. Product and gene sizes are given in base pairs (bp).

**Table 2:** RT qRT-PCR thermocycling protocol.

Cycle Step	Temperature	Time	Cycles
Reverse Transcription	55°C	10 min	1
Initial Denaturation	95°C	1 min	1
Denaturation	95°C	10 secs	40
Extension	62°C	30 secs + plate read	
Melt Curve	65°C to 90°C in 0.5°C increments	Increment every 5 secs + plate read	1

### 2.8 Statistical analysis

**Figure 1:**
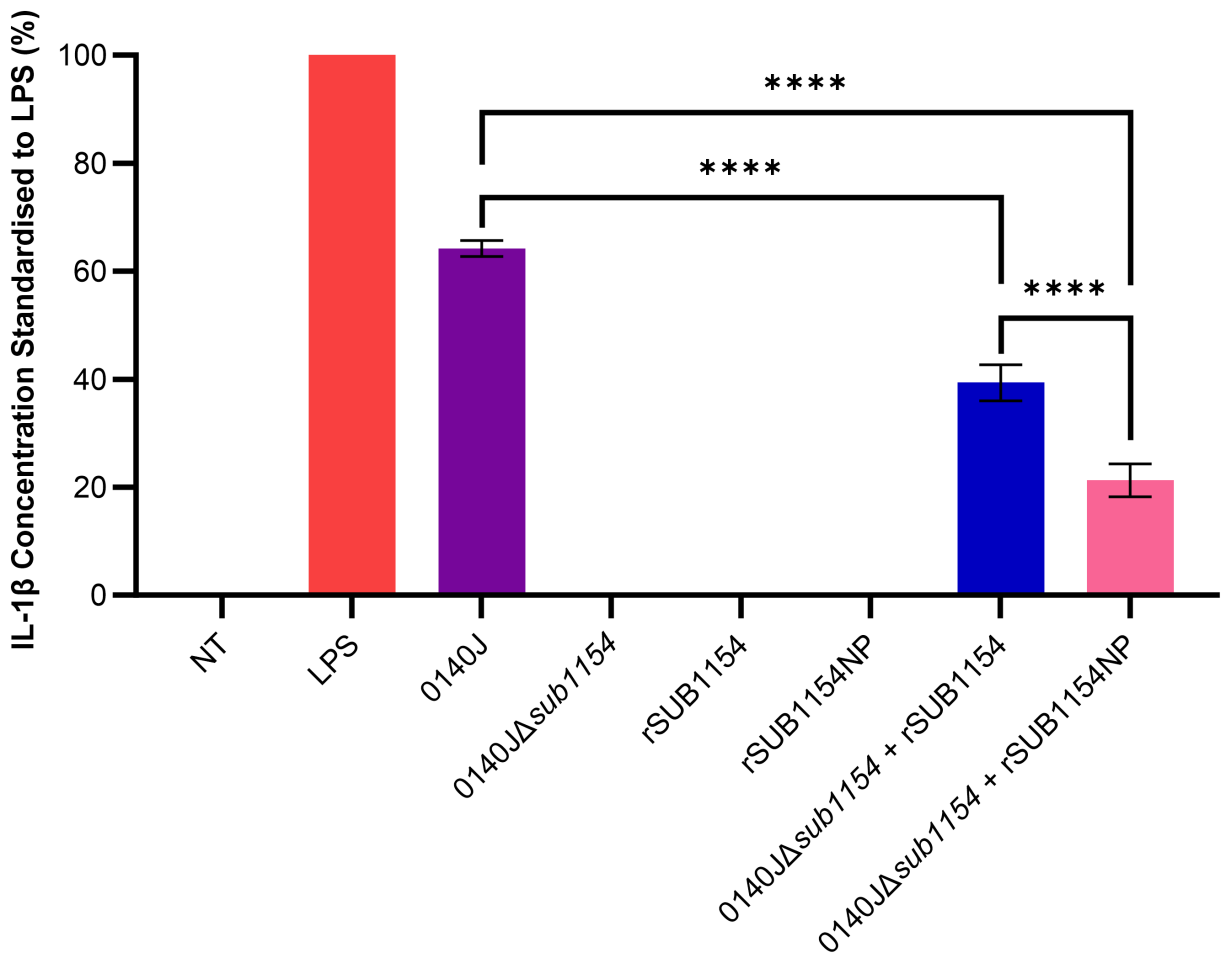
*S. uberis* SUB1154 protein is involved in the production of IL-1β from BMMOs. Bovine mammary macrophages (BMMOs) were isolated from milk and seeded into culture dishes at 50,000 BMMOs/well. BMMOs were challenged with either heat-killed *S. uberis* strain 0140J or SUB1154 deletion mutant (0140JΔ*sub1154*) at a multiplicity of infection (MOI) of 50:1 bacterium:BMMO and/or 2 nM rSUB1154 or rSUB1154NP (proteolytically compromised) protein. Supernatants were collected 20h after challenge and the concentration of IL-1β was measured by ELISA. BMMOs were unstimulated in the no treatment (NT) group and this mean was deducted from the other values, which were then standardised to the LPS (10 ng/mL) positive control. Data is presented as N=3 ± SD. Data was statistically analysed using a one-way ANOVA followed by Tukey multiple comparisons *post hoc* test (****P<0.0001).

**Figure 2:**
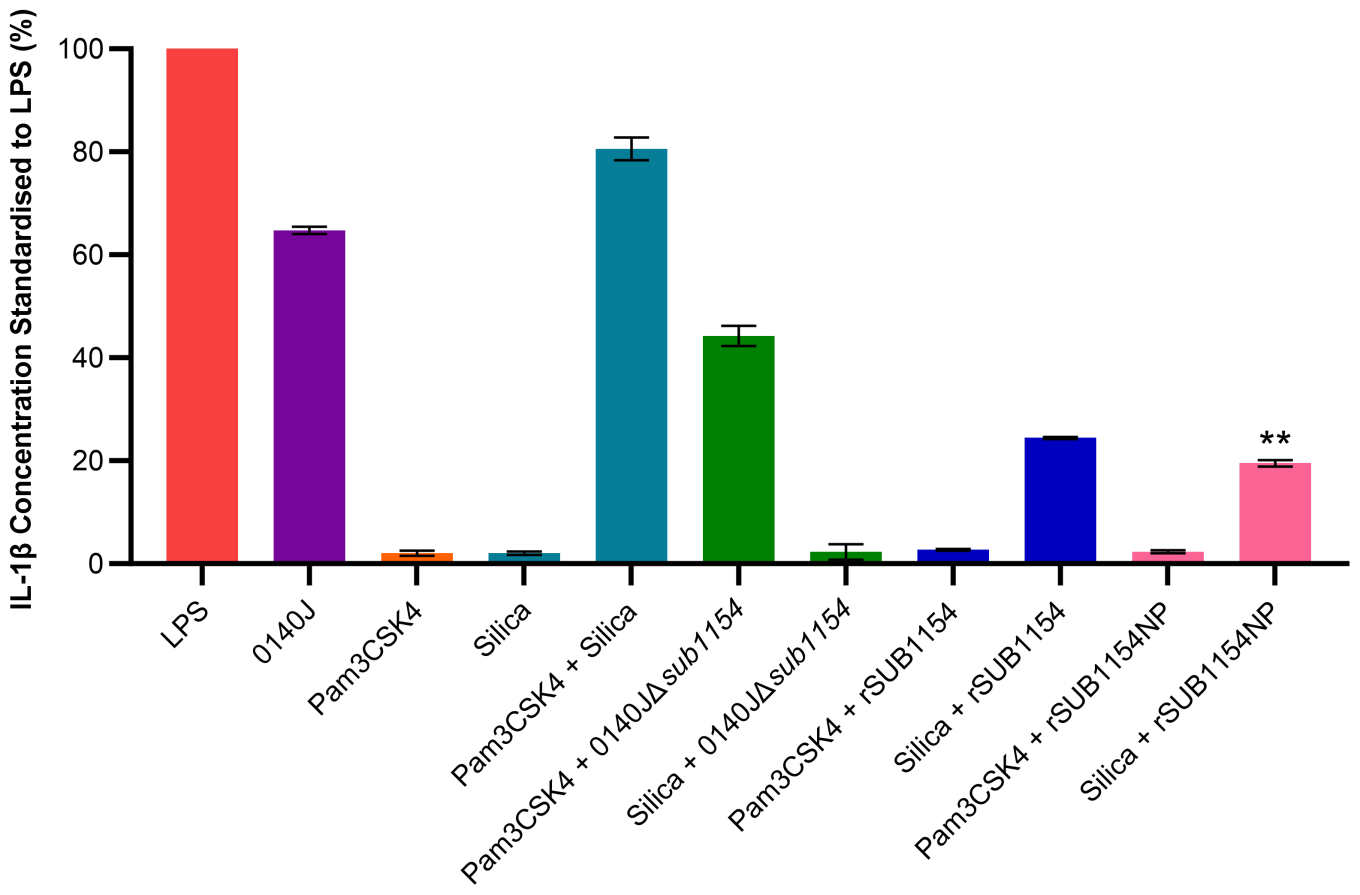
*S. uberis* SUB1154 protein primes the NLRP3 inflammasome in BMMOs. Bovine mammary macrophages (BMMOs) at 50,000 BMMOs/well were challenged with heat-killed *S. uberis* strain 0140J or SUB1154 deletion mutant (0140JΔ*sub1154*) at a multiplicity of infection (MOI) of 50:1 bacterium:BMMO and/or 2 nM rSUB1154 or rSUB1154NP (mutant predicted protease site) protein. Supernatants were collected 20h after challenge and the concentration of IL-1β measured by ELISA. BMMOs were unstimulated in a no treatment group and the mean of this group was deducted from the other values and standardised to the LPS positive control (10 ng/mL). Data is presented as N=3 ± SD. In addition to *S. uberis* and rSUB1154 and NP proteins, BMMOs were challenged with 1.0 µg/mL Pam3CSK4 (primes the inflammasome) and 500 µg/mL silica (activates the inflammasome). Data was statistically analysed using a one-way ANOVA followed by Tukey multiple comparisons *post hoc* test (**P<0.01 compared to silica + rSUB1154).

**Figure 3:**
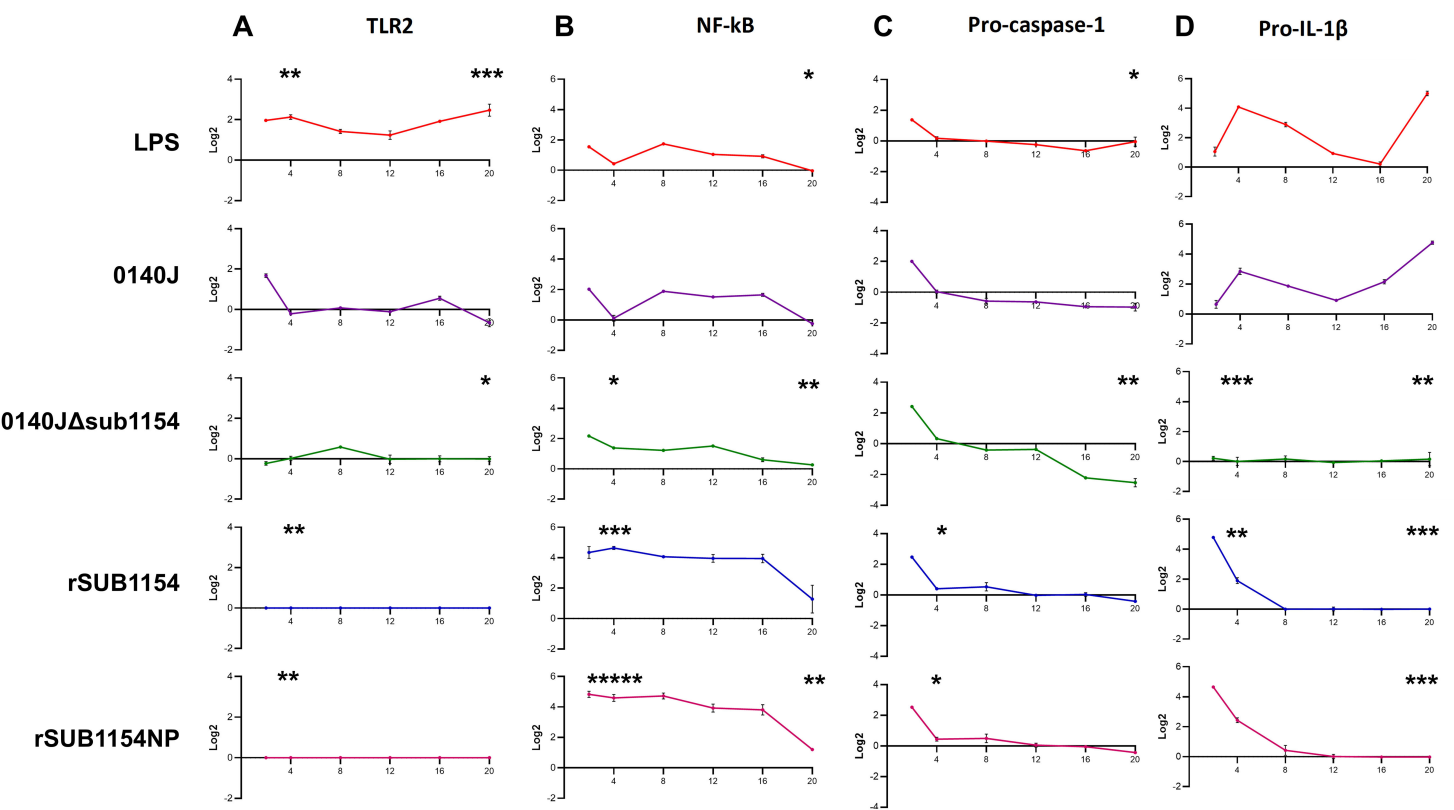
BMMO differential RNA expression of inflammasome pathway genes in response to *S. uberis* stimulation. RNA was extracted from isolated bovine mammary macrophages (BMMOs) at 0, 2, 4, 8, 12, 16 and 20h after challenge with either 10 ng/mL LPS; heat-killed *S. uberis* strain 0140J or SUB1154 deletion mutant (0140JΔ*sub1154*) at a multiplicity of infection (MOI) of 50:1 bacterium:BMMO; 2 nM rSUB1154 or rSUB1154NP (proteolytically compromised) protein. Changes in mRNA quantity were determined by real time reverse transcription quantitative PCR using 3 reference genes (GAPDH, ACTB and RPL13a) for 4 target genes: TLR2 **(A)**, NF-kB **(B)**, pro-caspase-1 **(C)** and pro-IL-1β **(D)**. Log_2_ was calculated and the data is presented as N=3 ± SD. Values at 0h were used to determine baseline mRNA levels (0 Log_2_). Asterisks denote significance calculated with Two-Way ANOVA and Dunnett’s multiple comparisons test, compared to 0140J, at 4h and 20h; * *P<*0.05, ** *P<*0.01,*** *P<*0.001 **** *P<*0.0001. All timepoint statistics available in **Supplementary Table 1**.

**Figure 4:**
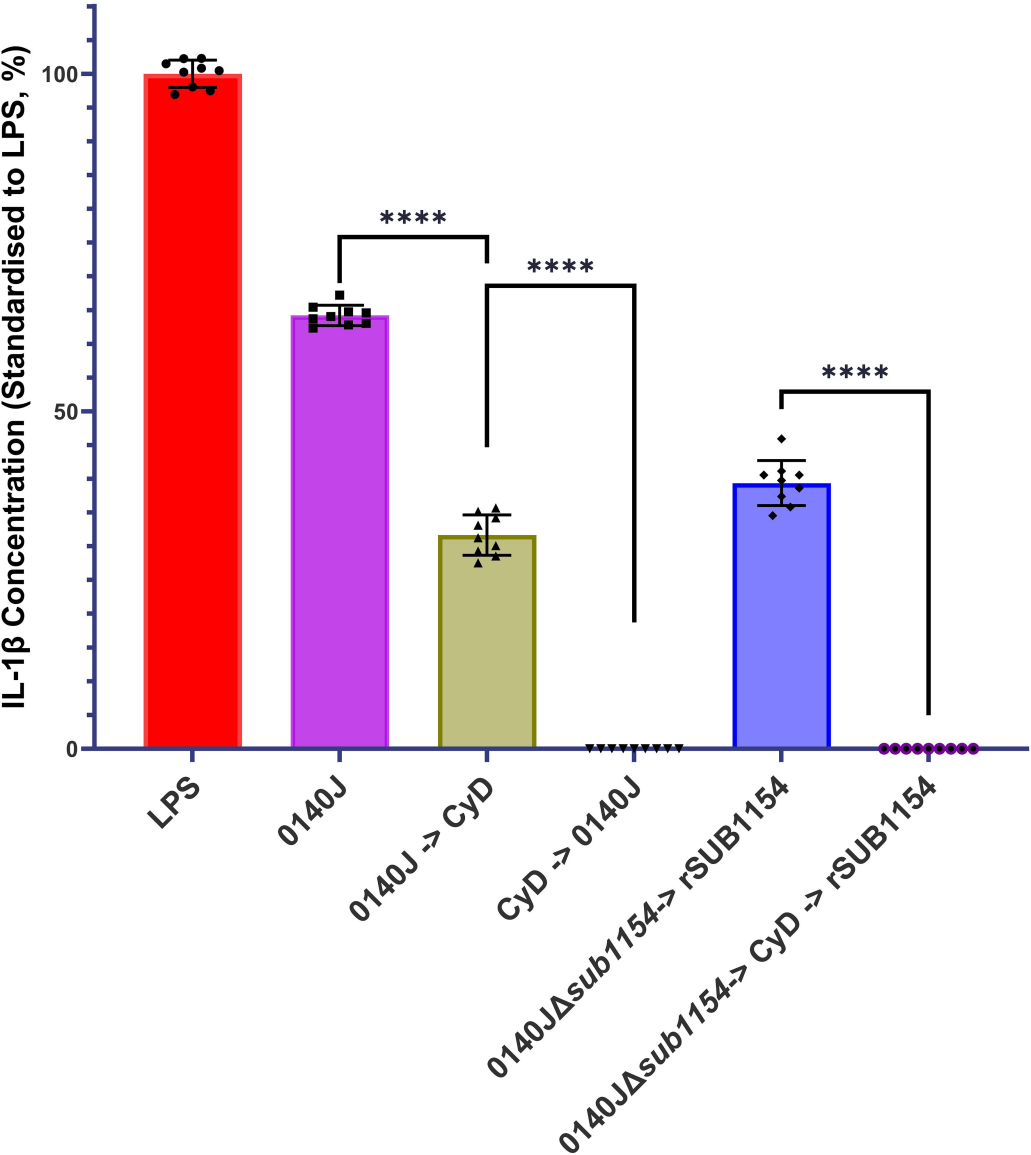
*S. uberis* SUB1154 protein functions intracellularly to prime the NLRP3 inflammasome in BMMOs. Bovine mammary macrophages (BMMOs) were isolated from milk and seeded into culture dishes at 50,000 BMMOs/well. BMMOs were challenged with either heat-killed *S. uberis* strain 0140J or SUB1154 deletion mutant (0140JΔ*sub1154*) at a multiplicity of infection (MOI) of 50:1 bacterium:BMMO and/or 2 nM rSUB1154 protein. Cell entry was inhibited by incubating BMMOs with 10 µM Cytochalasin D (CyD) for 2h. Supernatants were collected 20h after challenge and the concentration of IL-1β was measured by ELISA. BMMOs were unstimulated in a no treatment group and the mean of this group was deducted from the other values and standardised to the LPS positive control (10 ng/mL). Data is presented as N=9 ± SD. Data was statistically analysed using a one-way ANOVA followed by Tukey multiple comparisons *post hoc* test ****=p<0.0001.

**Figure 5:**
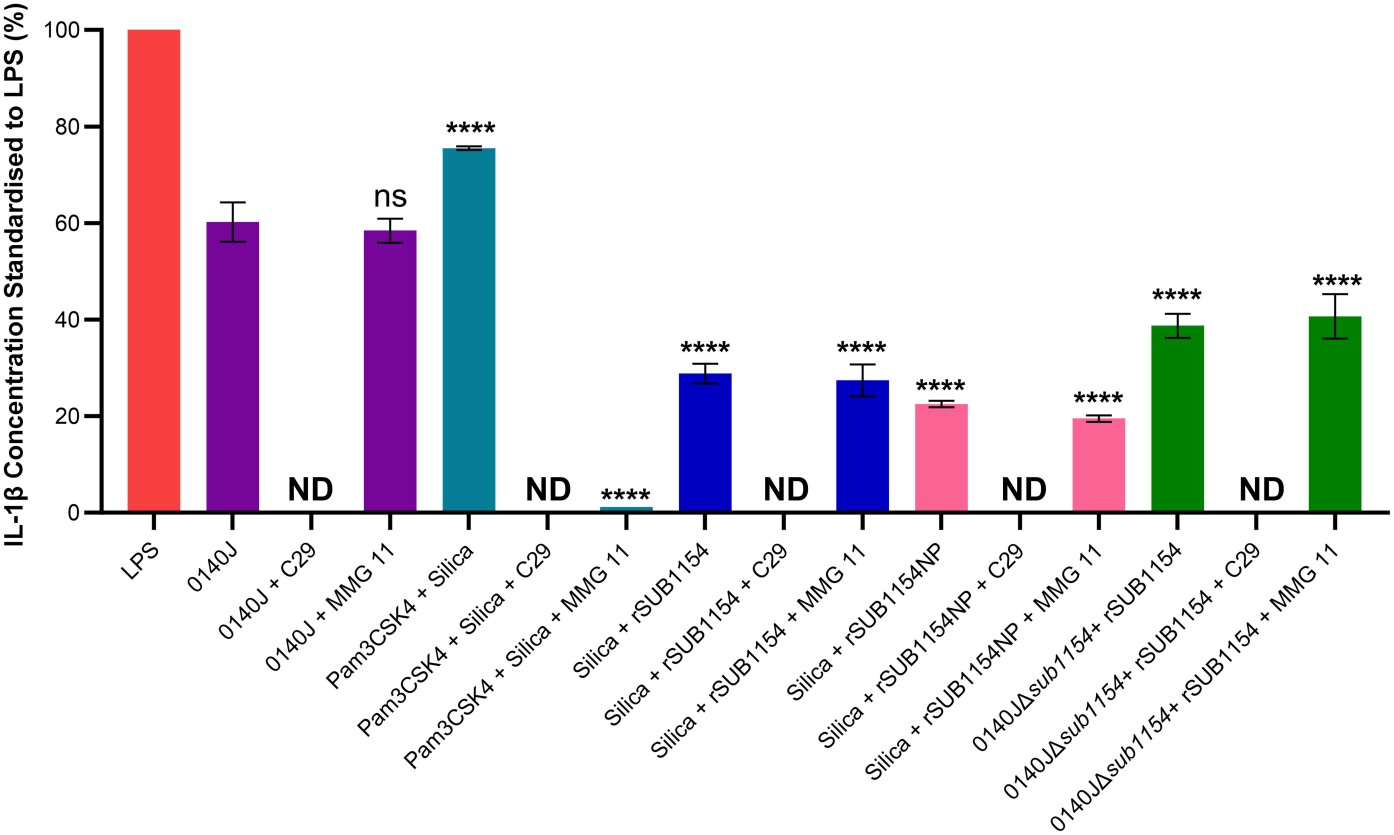
SUB1154 primes the inflammasome by interacting with intracellular TIR domains. Bovine mammary macrophages (BMMOs) were isolated from milk and seeded into culture dishes at 50,000 BMMOs/well. BMMOs were incubated in the presence and absence of the TLR2 inhibitors C29 (intracellularly binds to the TIR (toll-interleukin receptor) domain; 100 µM) or MMG 11 (antagonist to extracellular binding of TLR2; 100 µM) 1h prior to subsequent challenge with either heat-killed *S. uberis* strain 0140J or SUB1154 deletion mutant (0140JΔ*sub1154*) at a multiplicity of infection (MOI) of 50:1 bacterium:BMMO and/or 2 nM rSUB1154 or rSUB1154NP (proteolytically compromised) protein; 1.0 µg/mL Pam3CSK4 (primes the inflammasome) and/or 500 µg/mL silica (activates the inflammasome). IL-1β concentration was measured from the supernatants after 20h by ELISA and values were standardised to LPS. Data is presented as N=3 ± SD, statistically analysed using a one-way ANOVA followed by Tukey multiple comparisons *post hoc* test ****=p<0.0001, ns, not significant, comparisons against 0140J alone. ND, none detected.

Data was analysed using GraphPad Prism 10.0.3. Data was statistically analysed using either a one-way ANOVA followed by Tukey multiple comparisons *post hoc* test (**Figures 1**–**4**) or a two-way ANOVA followed by Dunnett’s multiple comparisons *post hoc* test (**Figure 5**). A value of *P ≤* 0.05 was considered to indicate a statistically significant difference.

The RT qRT-PCR data was analysed by first determining the mean Cq value for each condition using the three reference genes (ACTB, GAPDH, RPL13a). Then, for each condition, the mean Cq was deducted from the corresponding test gene Cq to determine the ΔCq. The ΔCq was then averaged for the no treatment condition (one value for each test gene). After that, the ΔΔCq was determined by deducting the mean no treatment ΔCq from each of the conditions. From these values, the 2^-ΔΔCq (Relative Quantification, RQ) was calculated. Finally, the log-2 was calculated for all the RQ values. Values at 0h were used to determine baseline transcription (0 Log_2_).

## 3 Results

### 3.1 SUB1154 is involved in the BMMO inflammatory response to *S. uberis*

Previous research has shown that the SUB1154 protein is involved in the inflammatory response to *S. uberis* in *ex-vivo* bovine mammary macrophage extracted from milk (BMMO) (Archer et al., 2020). Here, we deployed this model, challenged BMMOs with different stimuli and determined the IL-1β concentration by ELISA, standardised to that obtained in response to LPS, as previously described (Tomes et al., 2024) (**Figure 1**). BMMOs were responsive to LPS or *S. uberis* but in the absence of challenge (NT) we detected no IL-1β. Similarly, in the absence of SUB1154 (using a deletion mutant; 0140JΔ*sub1154*), there was no IL-1β produced. Recombinant SUB1154 alone also did not elicit a response, with the predicted protease domain intact (rSUB1154) or altered (rSUB1154NP). When BMMOs were challenged with both the rSUB1154 and deletion mutant, there was restoration of IL-1β production, however, this was significantly less when compared to treatment with the genetically intact parental strain, 0140J (*P<*0.0001). Treatment with rSUB1154NP and deletion mutant also restored IL-1β production but at a significantly lower level compared to that obtained with rSUB1154 and deletion mutant (*P<*0.0001) (**Figure 1**).

### 3.2 SUB1154 primes the inflammasome

Next, we sought to unpick the target of SUB1154. Inflammasome activity is controlled in a two-stage process, priming and activation. Here, we exploit molecules which specifically prime or activate the inflammasome to determine the stage at which SUB1154 acts. We chose Pam3CSK4 which can prime the NLRP3 inflammasome but not activate it, and silica which activates the NLRP3 inflammasome but does not prime it. BMMOs were challenged in various combinations with 0140J, 0140JΔ*sub1154*, rSUB1154, rSUB1154NP, Pam3CSK4 and silica and the IL-1β production determined by ELISA (**Figure 2**; **Supplementary Table 1**). BMMOs challenged with 0140JΔ*sub1154* and the inflammasome primer Pam3CSK4, produced IL-1β, but when challenged with 0140JΔ*sub1154* and the inflammasome activator, silica, there was minimal IL-1β production. Conversely, BMMOs challenged with rSUB1154 or rSUB1154NP and Pam3CSK4 produced minimal IL-1β, but when challenged with either rSUB1154 or rSUB1154NP and silica BMMO produced IL-1β. There was significantly less IL-1β produced from BMMOs activated with silica and primed with rSUB1154NP compared to silica and rSUB1154 (*P<*0.01). Therefore, SUB1154 appears to function in a manner consistent with the activity of Pam3CSK4; a primer of the inflammasome.

### 3.3 SUB1154 primes the inflammasome downstream of TLR2 but upstream of NF-κB

It has remained unclear which host machinery triggers the responses to *S. uberis*, but existing work has demonstrated that it does not happen via the cell surface receptor domain of TLR2 (Farhat et al., 2008). Thus, we sought to determine the target of the SUB1154 protein. To do this, we performed real-time quantitative reverse transcription PCR. (RT-qRT-PCR) for a panel of NLRP3-related genes (TLR2, NF-kB, pro-IL-1β and pro-caspase-1) on RNA extracted from BMMOs following challenge with LPS, *S. uberis* stain 0140J, 0140JΔ*sub1154*, rSUB1154 or rSUB1154NP (**Figure 3**). We did this across a time-course of 2-20 hours.

TLR2 exhibited baseline expression levels following treatment with 0140JΔ*sub1154* and rSUB1154/NP, whereas treatment with LPS consistently elevated TLR2 mRNA levels. Stimulation with 0140J increased TLR2 mRNA levels briefly with a small peak at 2h, returning to baseline at 4-12h, with a small increase again at 16h with a decreased expression at 20h (**Figure 3A**).

Broadly, LPS and 0140J induced similar changes in measured mRNAs, with increased NF-κB mRNA levels at 2h returning to baseline by 4h. This was followed by increased mRNA abundance at 8h that gradually returned to baseline expression at 20h. The deletion mutant 0140JΔ*sub1154* initially induced similar mRNA expression of NF-κB as LPS and 0140J, however, there was no return to baseline at 4h and instead mRNA peaked at 2h before gradually decreasing to baseline expression at 20h. On the other hand, at 2h post treatment with rSUB1154/NP twice the expression of NF-κB was detected compared to that in response to LPS, 0140J or 0140JΔ*sub1154*. This elevated expression level persisted to 16h before decreasing to near baseline expression at 20h (**Figure 3B**).

Treatment with either LPS or 0140J induced changes in pro-IL-1β with log_2_fold-changes (l2FC) of 4 and 3 by 4h respectively, followed by a steady decrease until 16h (LPS) and 12h (0140J). This was followed by an increase in pro-IL-1β levels that peaked at l2FC of 5 by 20h for treatments with LPS and 0140J. Treatment with 0140JΔ*sub1154* did not alter pro-IL-1β levels, which remained at baseline for the duration of the study. Stimulation with rSUB1154/NP induced an enrichment of pro-IL-1β mRNA by 2h, which returned to baseline by 8h (**Figure 3C**).

LPS, 0140J, 0140JΔ*sub1154* and rSUB1154/NP induced similar changes in pro-capspase-1 mRNA levels. There was a peak in mRNA levels at 2h which returned to baseline by 4h. Afterwards, baseline expression was maintained in the presence of LPS and rSUB1154/NP. Treatment with 0140J and 0140JΔ*sub1154* decreased pro-caspase-1 mRNA levels to l2FC of -1 at 8h, which was maintained throughout the study for 0140J treatment. There was a further decrease in pro-caspase-1 levels with 0140JΔ*sub1154* treatment to L2FC of -2.5 by 16h (**Figure 3D**).

### 3.4 SUB1154 functions intracellularly in BMMOs

Prior work suggested that SUB1154 has a role in the NLRP3 inflammasomal pathway leading to IL-1β secretion (Archer et al., 2020). However, it is not clear if SUB1154 functions extracellularly or intracellularly. We challenged BMMOs with *S. uberis* strain 0140J in the presence and absence of the cell entry inhibitor, Cytochalasin D (CyD) and measured IL-1β secretion (**Figure 4**). Inhibition of BMMO uptake prior to challenge ablated IL-1β secretion, but a reduction was also observed if CyD followed challenge. Next, we asked if the SUB1154 protein itself needed to be internalised for this response. To test this, we exploited the 0140JΔ*sub1154* strain in combination with CyD and recombinant SUB1154 protein. Challenging with 0140JΔ*sub1154*, before blocking cell uptake with CyD, and then subsequently adding rSUB1154, prevented IL-1β secretion.

### 3.5 SUB1154 primes the inflammasome through an intracellular TIR domain

IL-1β secretion is an end-product of the priming and activation of the NLRP3 inflammasome. TLR2 is the receptor commonly associated with NLRP3-mediated inflammasome responses to many Gram-positive pathogens, yet it does not respond to *S. uberis* (Farhat et al., 2008). However, studies examining TLR2 do so under the canon that it is the extracellular domain of TLR2, found on the cell surface, that binds to PAMPs. We returned to this assumption considering the now clear evidence (**Figure 3**) for an intracellular interaction and the changes in TLR2 mRNA level detected (**Figure 5**). To do this, we challenged BMMOs following treatment with a compound which modulates TLR2 binding (**Figure 4**). This confirmed that our BMMO model interacts canonically with TLR2 inhibitors as both C29 and MMG 11 prevented IL-1β production upon challenge with Pam3CSK4 and silica. As expected, (e.g. Farhat et al., 2008) extracellular binding to TLR2 inhibition by the antagonist MMG 11 did not prevent or reduce IL-1β secretion in response to *S. uberis*. However, when the intracellular interaction between the TLR2-TIR (toll-interleukin receptor) domain and MyD88 was inhibited by C29, *S. uberis* was unable to elicit an inflammasome response. Further, pretreatment with C29, but not pretreatment with MMG 11, prevented an IL-1β response by BMMOs in response to challenge with rSUB1154 and silica. Similarly, pretreatment with C29 ablated IL-1β production from BMMOs 0140JΔ*sub1154* and rSUB1154 whereas pretreatment with MMG 11 had no effect. Together, these show that SUB1154 indeed acts on intracellular components of TLR2.

## 4 Discussion

*S. uberis* infection of the bovine mammary gland has an unusual pathology in which the host inflammatory response benefits, rather than hinders, the pathogen. Here, we dissect the host-pathogen interactions at the earliest stages of colonisation. We confirm that the *S. uberis* SUB1154 protein plays a key role in this host-pathogen interaction by modulating the NLRP3 inflammasomal pathway. Utilising well-characterised triggers for priming and activation alongside our previously developed (Archer et al., 2020; Tomes et al., 2024) *in vitro* model, we demonstrate that the SUB1154 protein primes the inflammasome without directly activating it. We then go on to show that IL-1β production by BMMOs challenged with *S. uberis* is dependent upon SUB1154, with deletion of the SUB1154 protein from the bacteria (0140JΔ*sub1154*) ablating the IL-1β response from isolated BMMOs (**Figure 1**). Paradoxically, 0140JΔ*sub1154* strain is less pathogenic *in vivo* (Archer et al., 2020), which we previously showed was due to its reduced ability to trigger an inflammatory response. Here, we were able to complement the response to 0140JΔ*sub1154* with recombinant SUB1154 protein and demonstrate that the inflammasome response is dependent upon its uptake into macrophages (**Figure 3**). This contrasts Gram-positive canonical pathology in which it is TLR2 found on the extracellular membrane that responds to bacteria and triggers the MyD88-NLRP3 downstream signalling pathway to prime the inflammasome.

**Figure 6:**
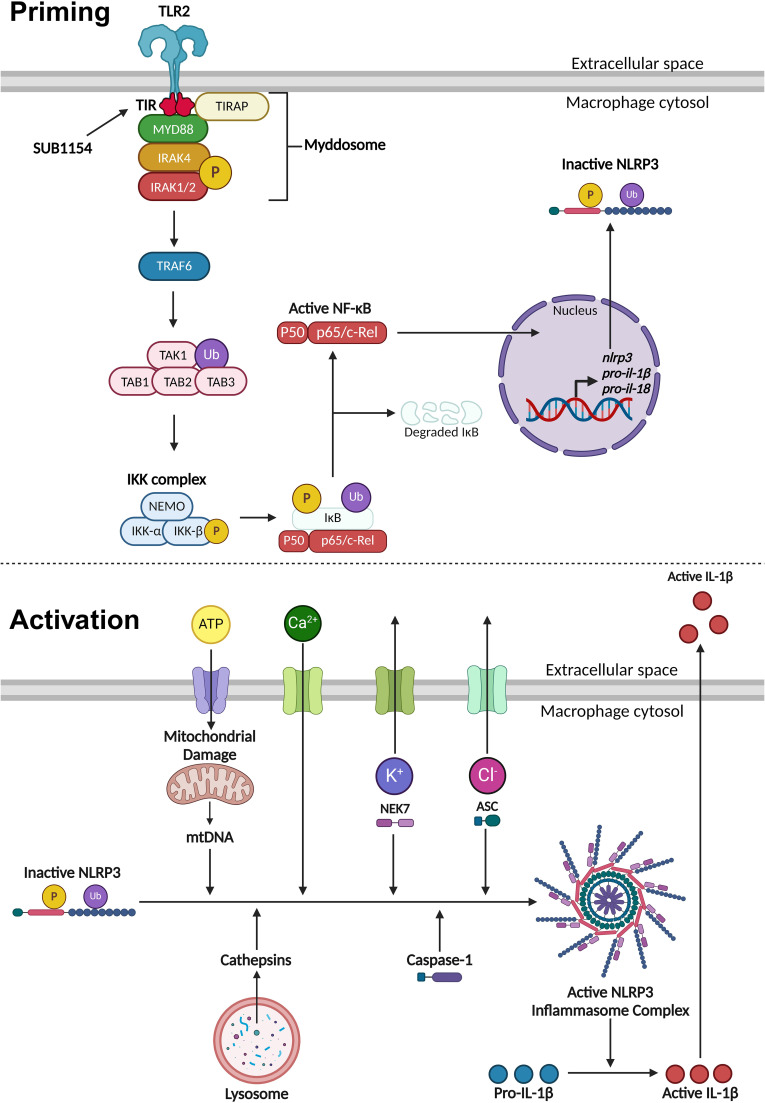
Priming and activation of the NLRP3 inflammasome. NLRP3 activity requires a priming and activation signal. *S. uberis* primes the NLRP3 inflammasome by the SUB1154 protein interacting with the intracellular domain of TLR2 expressed on the extracellular membrane of macrophages. This causes a downstream cascade where the TLR2-TIR domain dimerises. TIRAP associates with the plasma membrane and the TLR2-TIR domain, forming a bridge to allow the MyD88 adaptor protein to associate and activate. MyD88 recruits IRAK4 which autophosphorylates (P), subsequently activating IRAK1/2 and creating a large signalling complex called the Myddosome. IRAK1 activates TRAF6, which ubiquitinates (Ub) TAK1 to form a complex with TAB1, 2 and 3, activating TAK1. This complex activates the IKK complex consisting of three subunits: NEMO, IKKα and IKKβ. The activated IKK complex phosphorylates and ubiquitinates IκB causing it to dissociate from NF-κB. Active NF-κB translocates into the nucleus and causes increased transcription of *nlrp3* and inactive pro forms of the pro-inflammatory cytokines *il-1β* and *il-18*. Once NLRP3 is primed, the inflammasome can become assembled and activated. This can occur through a variety of different stimuli. Phagocytosed compounds can be trafficked to the lysosome where they cause the release of cathepsins. Extracellular ATP can enter the cytosol and cause mitochondrial damage resulting in the release of mtDNA that interacts with NLRP3. Calcium ion influx promotes NLRP3 inflammasome assembly. Potassium ion efflux causes NLRP3 oligomerisation in a NEK7 dependent manner. Chloride ion efflux induces ASC polymerisation. Activated NLRP3 inflammasome complex allows caspase-1 to cleave pro-IL-1β into its active form which is secreted out of the macrophage to initiate an immune response. Figure created with BioRender.com.

NLRP3 inflammasome activity is controlled in two stages, priming and activation (**Figure 6**). The priming step is needed to increase gene and protein expression of inflammasome components, after which NLRP3 is maintained in an inactive state through a variety of post-translational modifications (Yang et al., 2017; Swanson et al., 2019; Duez and Pourcet, 2021). The NF-κB transcription factor supports this drastic remodelling of gene expression following its translocation into the nucleus (Yang et al., 2017; Gong et al., 2020; Duez and Pourcet, 2021). Unusually, *S. uberis* infection of the mammary gland is enhanced by the host immune response but does not elicit responses from the mammary epithelium (or during its normal commensal existence in other niches) (Günther et al., 2016a; Archer et al., 2020). We previously demonstrated that while *S. uberis* is resistant to the effects of the inflammatory response *in vivo*, the increased metabolism and host damage in the local area likely increases nutrient availability for the growing bacteria (Archer et al., 2020).

Once the inflammasome receives both priming and activating signals, pro-IL-1β is cleaved into its active form and is secreted out of the BMMO to initiate an immune response (Yang et al., 2017; Swanson et al., 2019; Duez and Pourcet, 2021). Our *in vitro* model enabled us to dissect this process in isolated BMMOs. Here, we used combinations of chemical compounds, small molecular inhibitors, recombinant SUB1154 protein, and isogenic *S. uberis* strains. We found that IL-1β was secreted by BMMOs following stimulation with rSUB1154 and silica, a compound which activates the inflammasome, but not when challenged with rSUB1154 and Pam3CSK4, a compound which primes the inflammasome. Silica beads stimulate lysosomal degradation resulting in increased cytosolic cathepsin B which interacts with NLRP3 promoting activation of caspase-1 to cleave pro-IL-1β into its active form (Chevriaux et al., 2020). Alternatively, Pam3CSK4 is a TLR2 agonist that triggers downstream signalling providing the NLRP3 priming signal (Brandt et al., 2013). Secretion of IL-1β from BMMOs was not present when stimulated by Pam3CSK4 alone or in combination with SUB1154 but was restored when Pam3CSK4 priming was complemented with either silica or the *S. uberis* mutant (0140JΔ*sub1154*), which lacks SUB1154. Together, these data strongly suggest that SUB1154 provides the priming signal and that other components of the *S. uberis* bacterial cell, or responses of the BMMOs to the bacterium, generate the activation signal for inflammasome activation.

Here, we have shown that uptake of the bacteria is required for the inflammatory response, as IL-1β release was absent when bacterial entry was blocked by the endocytosis inhibitor Cytochalasin D (CyD), but CyD did not prevent IL-1β release from BMMOs in which *S. uberis* had already been taken up (**Figure 3**). Surprisingly, the priming step of inflammasome activation (interaction with SUB1154) was shown to be sensitive to inhibition with CyD (**Figure 3**); implying that the priming step may occur at an intracellular location. Canonically, TLR2 is the PRR stimulated by Gram-positive bacteria. However, while MMG 11 prevents Pam3CSK4 interacting with TLR2 *in vitro* (**Figure 4**), it did not prevent SUB1154 from priming the inflammasome nor did it prevent inflammasome activation by intact *S. uberis*. MMG 11 acts as a competitive antagonist that displaces Pam3CSK4 (Grabowski et al., 2018, 2020) preventing interactions with the extracellular domains of TLR2. This is concordant with other works examining the role of TLR2 in *S. uberis* interactions with the host. For example, human embryonic kidney 293 cells transfected with whole bovine TLR2 showed no response to *S. uberis* (Farhat et al., 2008). Similarly, response from a primary culture of bovine MECs showed no response following challenge with *S. uberis* (Günther et al., 2016a). Whilst isolated lipoteichoic acid (LTA) from *S. uberis* has been shown to induce a strong immune response in bMECs, this was not through the TLR2 pathway (Günther et al., 2016a). These data are consistent with our finding implying that *S. uberis* is not recognised by the extracellular domain of TLR2. The existing data also further demonstrates that LTA bound to the bacterium is inaccessible to its target host. This explains the absence of immune recognition of this bacterium by epithelial tissue during its commensal existence and within the bovine mammary gland (Günther et al., 2016b). However, another TLR2 inhibitor, C29 inhibited SUB1154 mediated inflammasome responses and that to the intact bacterium. C29 binds within the BB loop of the toll-interleukin receptor (TIR) domain – an intracellular region of the protein (Mistry et al., 2015). Taken together, our data therefore strongly imply that the SUB1154 priming signal occurs at a site other than the recognised TLR2 receptor and that priming by TLR activation still requires a productive interaction between the TLR2-TIR domain and MyD88.

Bacteria possess TIR domains that are thought to target mammalian TIR complexes (Lee et al., 2022). Studies have mostly focused on the potential role of bacterial TIR proteins as virulence factors that inhibit host TLR signalling. For example, TcpC is a TIR homologous protein identified in uropathogenic *E. coli* that binds to TLR4 and MyD88 through TIR domain interactions to inhibit downstream signalling (Yadav et al., 2010; Rana et al., 2012). TcpC knockout strains show reduced ability to survive in macrophages (Cirl et al., 2008). However, there is evidence that the bacterial TIR, TlpA, from *Salmonella enteric* serovar Enteritidis stimulates the host inflammasomal pathway as this protein activates caspase-1, resulting in cleavage and secretion of IL-1β. Macrophages infected with wild-type *S. enterica* serovar Enteritidis produce an increase in IL-1β compared to stimulation with the *tlpA* deletion strain (Newman et al., 2006). These studies demonstrate bacterial-TIR interactions are not unique and may have various consequences on the inflammasomal pathway. The interaction(s) between SUB1154 and the TLR2-TIR domain in BMMOs postulated here merits further detailed investigation alongside the role of similar cell envelope serine proteases of pyogenic streptococci in the inflammasome pathway in cells from their target species.

## 5 Conclusion

Our data indicate that *S. uberis* does not interact with ligand binding domain of TLR2 expressed on the extracellular surface of BMMOs. Instead, *S. uberis* is internalised by BMMO from where SUB1154 primes the NLRP3 inflammasome by a mechanism that requires the TLR-TIR domain. This interaction provides downstream signalling allowing other factors attributed to the bacterial cell to provide the activation signal. Together, these signals allow for NLRP3 inflammasome activity to secrete active pro-inflammatory cytokines, such as IL-1β, to initiate an immune response, precipitating the inflammatory response and ultimately disease pathology.

## Data Availability

The original contributions presented in the study are included in the article/**Supplementary Material**. Further inquiries can be directed to the corresponding author.

## References

[B1] Anderson E. T. Wetherell M. G. Winter L. A. Olmsted S. B. Cleary P. P. Matsuka Y. V. (2002). Processing, stability, and kinetic parameters of C5a peptidase from *Streptococcus pyogenes* . Eur. J. Biochem. 269, 4839–4851. doi: 10.1046/j.1432-1033.2002.03183.x 12354115

[B2] Archer N. Egan S. A. Coffey T. J. Emes R. D. Addis M. F. Ward P. N.. (2020). A paradox in bacterial pathogenesis: activation of the local macrophage inflammasome is required for virulence of *Streptococcus uberis* . Pathogens. 9, 997. doi: 10.3390/pathogens9120997 33260788 PMC7768481

[B3] Bauernfeind F. G. Horvath G. Stutz A. Alnemri E. S. MacDonald K. Speert D.. (2009). Cutting Edge: NF-κB activating pattern recognition and cytokine receptors license NLRP3 inflammasome activation by regulating NLRP3 expression. J. Immunol. 183, 787–791. doi: 10.4049/jimmunol.0901363 19570822 PMC2824855

[B4] Bradley A. J. Leach K. A. Breen J. E. Green L. E. Green M. J. (2007). Survey of the incidence and aetiology of mastitis on dairy farms in England and Wales. Vet. Rec. 160, 253–257. doi: 10.1136/vr.160.8.253 17322356

[B5] Brandt K. J. Fickentscher C. Kruithof E. K. O. de Moerloose P. (2013). TLR2 ligands induce NF-κB activation from endosomal compartments of human monocytes. PloS One 8, e80743. doi: 10.1371/journal.pone.0080743 24349012 PMC3861177

[B6] Chen N. Xia P. Li S. Zhang T. Wang T. T. Zhu J. (2017). RNA sensors of the innate immune system and their detection of pathogens. IUBMB Life. 69, 297–304. doi: 10.1002/iub.1625 28374903 PMC7165898

[B7] Chevriaux A. Pilot T. Derangère V. Simonin H. Martine P. Chalmin F.. (2020). Cathepsin B is required for NLRP3 inflammasome activation in macrophages, through NLRP3 interaction. Front. Cell Dev. Biol. 8. doi: 10.3389/fcell.2020.00167 PMC7162607 32328491

[B8] Cirl C. Wieser A. Yadav M. Duerr S. Schubert S. Fischer H.. (2008). Subversion of toll-like receptor signaling by a unique family of bacterial toll/interleukin-1 receptor domain-containing proteins. Nat. Med. 14, 399–406. doi: 10.1038/nm1734 18327267

[B9] Davies P. L. Leigh J. A. Bradley A. J. Archer S. C. Emes R. D. Green M. J. (2016). Molecular epidemiology of *Streptococcus uberis* clinical mastitis in dairy herds: Strain heterogeneity and transmission. J. Clin. Microbiol. 54, 68–74. doi: 10.1128/JCM.01583-15 26491180 PMC4702729

[B10] Duez H. Pourcet B. (2021). Nuclear receptors in the control of the NLRP3 inflammasome pathway. Front. Endocrinol. 12. doi: 10.3389/fendo.2021.630536 PMC7947301 33716981

[B11] Egan S. A. Ward P. N. Watson M. Field T. R. Leigh J. A. (2012). Vru (Sub0144) controls expression of proven and putative virulence determinants and alters the ability of *Streptococcus uberis* to cause disease in dairy cattle. Microbiol. (Reading). 158, 1581–1592. doi: 10.1099/mic.0.055863-0 PMC3541772 22383474

[B12] Farhat K. Sauter K. S. Brcic M. Frey J. Ulmer A. J. Jungi T. W. (2008). The response of HEK293 cells transfected with bovine TLR2 to established pathogen-associated molecular patterns and to bacteria causing mastitis in cattle. Vet. Immunol. Immunopathol. 125, 326–336. doi: 10.1016/j.vetimm.2008.05.026 18621422

[B13] Gong T. Liu L. Jiang W. Zhou R. (2020). DAMP-sensing receptors in sterile inflammation and inflammatory diseases. Nat. Rev. Immunol. 20, 95–112. doi: 10.1038/s41577-019-0215-7 31558839

[B14] Grabowski M. Murgueitio M. S. Bermudez M. Rademann J. Wolber G. Weindl G. (2018). Identification of a pyrogallol derivative as a potent and selective human TLR2 antagonist by structure-based virtual screening. Biochem. Pharmacol. 154, 148–160. doi: 10.1016/j.bcp.2018.04.018 29684378

[B15] Grabowski M. Murgueitio M. S. Bermudez M. Wolber G. Weindl G. (2020). The novel small-molecule antagonist MMG-11 preferentially inhibits TLR2/1 signalling. Biochem. Pharmacol. 171, 113687. doi: 10.1016/j.bcp.2019.113687 31678495

[B16] Günther J. Czabanska A. Bauer I. Leigh J. A. Holst O. Seyfert H. M. (2016a). *Streptococcus uberis* strains isolated from the bovine mammary gland evade immune recognition by mammary epithelial cells, but not of macrophages. Vet. Res. 47, 13. doi: 10.1186/s13567-015-0287-8 26738804 PMC4704416

[B17] Günther J. Koy M. Berthold A. Schuberth H. J. Seyfert H. M. (2016b). Comparison of the pathogen species-specific immune response in udder derived cell types and their models. Vet. Res. 47, 22. doi: 10.1186/s13567-016-0307-3 26830914 PMC4736154

[B18] Guo H. Callaway J. B. Ting J. P. Y. (2015). Inflammasomes: Mechanism of action, role in disease, and therapeutics. Nat. Med. 21, 677–687. doi: 10.1038/nm.3893 26121197 PMC4519035

[B19] Hill A. W. Finch J. M. Field T. R. Leigh J. A. (1994). Immune modification of the pathogenesis of *Streptococcus uberis* mastitis in the dairy cow. FEMS Immunol. Med. Microbiol. 8, 109–117. doi: 10.1111/j.1574-695X.1994.tb00432.x 8173550

[B20] Hossain M. Egan S. A. Coffey T. Ward P. N. Wilson R. Leigh J. A.. (2015). Virulence related sequences; insights provided by comparative genomics of *Streptococcus uberis* of differing virulence. BMC Genomics 16, 334. doi: 10.1186/s12864-015-1512-6 25898893 PMC4427978

[B21] LaRock D. L. Russell R. Johnson A. F. Wilde S. LaRock C. N. (2020). Group A Streptococcus infection of the nasopharynx requires proinflammatory signaling through the interleukin-1 receptor. Infect. Immun. 88, e00356–e00320. doi: 10.1128/IAI.00356-20 32719155 PMC7504964

[B22] Lee E. Redzic J. S. Nemkov T. Saviola A. J. Dzieciatkowska M. Hansen K. C.. (2022). Human and bacterial toll-interleukin receptor domains exhibit distinct dynamic features and functions. Molecules. 27, 4494. doi: 10.3390/molecules27144494 35889366 PMC9318647

[B23] Leigh J. A. Egan S. A. Ward P. N. Field T. R. Coffey T. J. (2010). Sortase anchored proteins of Streptococcus uberis play major roles in the pathogenesis of bovine mastitis in dairy cattle. Vet. Res. 41, 63. doi: 10.1051/vetres/2010036 20519112 PMC2898060

[B24] Leigh J. A. Field T. R. Williams M. R. (1990). Two strains of Streptococcus uberis, of differing ability to cause clinical mastitis, differ in their ability to resist some host defence factors. Res. Vet. Sci. 49, 85–87. doi: 10.1016/S0034-5288(18)31052-X 2382062

[B25] Mistry P. Laird M. H. W. Schwarz R. S. Greene S. Dyson T. Snyder G. A.. (2015). Inhibition of TLR2 signalling by small molecule inhibitors targeting a pocket within the TLR2 TIR domain. Proc. Natl. Acad. Sci. U.S.A. 112, 5455–5460. doi: 10.1073/pnas.1422576112 25870276 PMC4418912

[B26] Moyes K. Drackley J. K. Morin D. E. Rodriguez-Zas S. L. Everts R. Lewin H. A.. (2010). Mammary gene expression profiles during an inflammatory challenge reveal potential mechanisms linking negative energy balance with impaired immune response. Physiol. Genomics 41, 161–170. doi: 10.1152/physiolgenomics.00197.2009 20103698 PMC4073896

[B27] Newman R. M. Salunkhe P. Godzik A. Reed J. C. (2006). Identification and characterization of a novel bacterial virulence factor that shares homology with mammalian toll/interleukin-1 receptor family proteins. Infect. Immun. 74, 594–601. doi: 10.1128/IAI.74.1.594-601.2006 16369016 PMC1346628

[B28] Rana R. R. Zhang M. Spear A. M. Atkins H. S. Byrne B. (2012). Bacterial TIR-containing proteins and host innate immune system evasion. Med. Microbiol. Immunol. 202, 1–10. doi: 10.1007/s00430-012-0253-2 22772799

[B29] Rathinam V. A. K. Fitzgerald K. A. (2016). Inflammasome complexes: Emerging mechanisms and effector functions. Cell. 165, 792–800. doi: 10.1016/j.cell.2016.03.046 27153493 PMC5503689

[B30] Sharma D. Kanneganti T. D. (2016). The cell biology of inflammasomes: Mechanisms of inflammasome activation and regulation. J. Cell Biol. 213, 617–629. doi: 10.1083/jcb.201602089 27325789 PMC4915194

[B31] Smith A. J. Ward P. N. Field T. R. Jones C. L. Lincoln R. A. Leigh J. A. (2003). MtuA, a lipoprotein receptor antigen from *Streptococcus uberis*, is responsible for acquisition of manganese during growth in milk and is essential for infection of the lactating bovine mammary gland. Infect. Immun. 71, 4842–4849. doi: 10.1128/IAI.71.9.4842-4849.2003 12933824 PMC187302

[B32] Swanson K. V. Deng M. Ting J. P. Y. (2019). The NLRP3 inflammasome: Molecular activation and regulation of therapeutics. Nat. Rev. Immunol. 19, 477–489. doi: 10.1038/s41577-019-0165-0 31036962 PMC7807242

[B33] Tassi R. McNeilly T. N. Fitzpatrick J. L. Fontaine M. C. Reddick D. Ramage C.. (2013). Strain-specific pathogenicity of putative host-adapted and nonadapted strains of *Streptococcus uberis* in dairy cattle. J. Dairy Sci. 96, 5129–5145. doi: 10.3168/jds.2013-6741 23769372

[B34] Tomes A. Archer N. Leigh J. (2024). Reproducible isolation of bovine mammary macrophages for analysis of host pathogen interactions. BMC Vet. Res. 20, 96. doi: 10.1186/s12917-024-03944-w 38461248 PMC10924389

[B35] Valderrama J. A. Riestra A. M. Gao N. J. Larock C. N. Gupta N. Ali S. R.. (2017). Group A streptococcal M protein activates the NLRP3 inflammasome. Nat. Microbiol. 2, 1425–1434. doi: 10.1038/s41564-017-0005-6 28784982 PMC5750061

[B36] von Moltke J. Ayres J. S. Kofoed E. M. Chavarría-Smith J. Vance R. E. (2013). Recognition of bacteria by inflammasomes. Annu. Rev. Immunol. 31, 73–106. doi: 10.1146/annurev-immunol-032712-095944 23215645

[B37] Wang N. Liang H. Zen K. (2014). Molecular mechanisms that influence the macrophage m1-m2 polarization balance. Front. Immunol. 5. doi: 10.3389/fimmu.2014.00614 PMC4246889 25506346

[B38] Whiley D. Jolley K. Blanchard A. Coffey T. Leigh J. (2024). A core genome multi-locus sequence typing scheme for *Streptococcus uberis*: an evolution in typing a genetically diverse pathogen. Microb. Genom. 10, 1225. doi: 10.1099/mgen.0.001225 PMC10963912 38512314

[B39] Yadav M. Zhang J. Fischer H. Huang W. Lutay N. Cirl C.. (2010). Inhibition of TIR domain signalling by TcpC: MyD88-dependent and independent effects on *Escherichia coli* virulence. PloS Pathog. 6, e1001120. doi: 10.1371/journal.ppat.1001120 20886104 PMC2944809

[B40] Yang J. Liu Z. Xiao T. S. (2017). Post-translational regulation of inflammasome. Cell Molecu Immunol. 14, 65–79. doi: 10.1038/cmi.2016.29 PMC5214939 27345727

